# Differential effects of intermittent anaerobic exercise on haematological parameters during and outside Ramadan

**DOI:** 10.4102/jphia.v16i1.1260

**Published:** 2025-10-08

**Authors:** Nizar Lotfi, Mohammed Madani

**Affiliations:** 1 Department of Physical Activity and Sport, Normal Superior School (ENS), Hassan II University, Casablanca, Morocco

**Keywords:** Ramadan, intermittent exercise, hematology, athletes, fasting, oxidative stress, iron metabolism

## Abstract

**Background:**

Fasting and exercise induce variable physiological changes depending on exercise intensity and nutritional state. Ramadan fasting, with its specific dietary restrictions, presents a unique context for evaluating these effects.

**Aim:**

This study aimed to assess the effect of intermittent anaerobic lactic exercise on variations in haematological parameters in male university athletes.

**Setting:**

Healthy male university sports students, participated in the study, performing intermittent anaerobic lactic exercise before and during Ramadan fasting.

**Methods:**

Ten healthy male university sports students (mean age 18.7 ± 0.6 years) were tested before and after a 30-min basketball game, during Ramadan and in a non-fasting period, with leukocytes (three indicators), red blood cell (RBC) (three indicators) and platelets (two indicators) parameters measured. Data were analysed using two-way analysis of variance to evaluate the effects of fasting, exercise, and their interaction.

**Results:**

The main results of our study showed that lymphocyte percentages decreased (*p* < 0.005, –26.84%), granulocyte percentages decreased (*p* < 0.005, –4.09%), haematocrit decreased (*p* < 0.005, –20.05%), haemoglobin decreased (*p* < 0.005, –18.02%), and RBC count decreased (*p* < 0.005, –15.9%) with exercise during Ramadan fasting. However, the percentage of intermediate cells parameter (percentage of intermediate blood cells among white blood cells) increased (*p* < 0.005, +12.98%) with exercise during Ramadan fasting. No significant haematological variations were observed under normal conditions outside the Ramadan fast.

**Conclusion:**

Intermittent anaerobic exercise during Ramadan fasting significantly alters haematological parameters, suggesting that fasting should be considered in athletes training regimens. These findings are limited b the small sample size (*n* = 10).

**Contribution:**

This study provides a detailed description of the haematological changes induced by intermittent anaerobic lactic exercise during Ramadan fasting in young male athletes. The findings highlight significant alterations in erythrocyte and leukocyte indices compared with the non-fasting state and underscore the need to consider fasting status when planning training loads, recovery and nutrition strategies for athletes.

## Introduction

The study of the interaction between intermittent anaerobic lactic exercise and fasting during the month of Ramadan is crucial for male university athletes. This period presents unique challenges in terms of nutritional management and training, with potential impacts on haematological responses to exercise.^1^ Ramadan fasting involves significant dietary changes, which can affect haematological parameters. Previous research has shown varied effects on haematocrit (HCT) and haemoglobin (Hb) levels during fasting. Some studies report increases,^2^ decreases,^3^ or no changes^4^ in these parameters. In addition, fasting can influence leukocyte levels and other haematological elements,^5,6^ indicating complex physiological responses.

Intensive training also has substantial impacts on haematological parameters. High-intensity exercise can lead to changes in white blood cell (WBC) counts, with increases following maximal exercise and decreases following high-intensity interval training.^7,8^ Endurance training is associated with a decrease in various haematological indices because of increased plasma volume.^9,10^ High-intensity acute exercise can activate blood platelet (PLT) function,^11,12,13,14,15^ while regular long-term high-intensity exercise may reduce PLT activation.^13,16,17^ During Ramadan, the combination of fasting and exercise can lead to specific haematological responses. Some studies suggest an acute increase in red blood cell (RBC) count because of dehydration-induced haemoconcentration,^18,19^ while others find no significant changes in WBC or PLT counts.^3,16^ There is a notable decrease in post-Ramadan RBC count in some cases,^3^ and varied responses in WBC counts following exercise.^7^ Platelet responses during Ramadan vary, with some studies observing reductions after high-intensity aerobic exercise,^10,20^ and others finding stable levels.^9,21^ Despite these findings, there is a lack of detailed studies specifically examining the effects of intermittent anaerobic lactic exercise during Ramadan on haematological parameters in male university athletes. This gap highlights the need for in-depth examination to understand the physiological implications during this unique period.

This study aims to evaluate the effect of intermittent anaerobic lactic exercise and Ramadan fasting on variations in haematological parameters in male university athletes. Specifically, it seeks to assess how these factors interact to influence RBC, WBC, and PLT parameters, with the goal of establishing optimal training and nutritional strategies during the month of Ramadan.

**Hypothesis:** We hypothesise that the interaction between intermittent anaerobic lactic exercise and Ramadan fasting will significantly affect haematological parameters in male university athletes.

## Research methods and design

### Participants

The study sample comprised a total of 10 healthy male university sports students (mean age 18.72 ± 0.59 years, mean body mass index [BMI] 20.69 kg/m^2^ ± 1.54 kg/m^2^ [range: 18.50–24.40], mean height 1.78 m ± 0.07 m). These participants abstained from food and drink during daylight hours in Ramadan, consistent with an overnight fasting state before each blood draw. Strict exclusion criteria were applied to eliminate any participant taking medication, having a history of cardiovascular disease, being a smoker, suffering from hypertension with systolic and diastolic blood pressure greater than 140/90 mmHg, diagnosed with diabetes, having a BMI greater than 30 kg/m^2^, or having musculoskeletal lesions of any kind. The sample size (*n* = 10) was selected based on logistical constraints (laboratory capacity, participant availability) and is comparable to similar physiological studies in athletic populations, providing a representative sample of moderately trained university athletes.

All participants had been involved in a varied physical training programme for 5 months, including football, rugby, gymnastics and athletics. This programme included 3–4 training sessions per week and was integrated into their university curriculum in Physical Education (Degree in Physical Education and Sport [LE-EPS]). The students were recruited from a larger cohort of 300 students enrolled in the same academic programme. Their moderate training status – 3–4 sessions weekly – further justifies sample homogeneity, as each participant acts as his own control, increasing statistical power in the repeated-measures design.^20,22,23^

### Haematological parameters measured

Blood samples were collected by venous puncture between 09:00 and 11:00 (after overnight fasting), using sterile plastic containers containing an anticoagulant ethylenediaminetetraacetic acid tripotassium salt (EDTA K3). The following haematological parameters were analysed: RBC count, HCT, Hb level, mean corpuscular volume (MCV), mean corpuscular haemoglobin (MCH), mean corpuscular haemoglobin concentration (MCHC) and mean haemoglobin content (MHC).

The parameters measured for leukocytes included total WBC count and their subpopulations: polymorphonuclear cells – neutrophils (NEU), eosinophils (EOS) – as well as lymphocytes (LYMP), monocytes (MON), intermediate cells (MID) and granulocytes (Gran). Platelet counts were also determined.

### Study design

This study employed a within-subject, repeated-measures design to evaluate the effects of Ramadan fasting on haematological responses to intermittent anaerobic lactic exercise. Participants underwent two experimental conditions: (1) a 30-min standardised basketball match performed 1 week before Ramadan (non-fasting period) and (2) a second identical match during the third week of Ramadan (fasting period). The basketball protocol was designed to replicate the intermittent, high-intensity demands of team sports, combining anaerobic lactic efforts (sprints, jumps) and aerobic endurance (continuous movement). Matches were structured as 5 versus 5 on a regulation court (28 m × 15 m), with enforced player rotations every 5 min to ensure balanced participation and workload distribution. Exercise intensity was monitored in real time using heart rate monitors (Polar Electro H10, Kempele, Finland) to maintain a target range of 75% – 90% of maximum heart rate (mean: 158 ± 12 beats per minute [bpm]), while external load metrics (distance covered, accelerations, sprint frequency) were quantified via Global Positioning system (GPS) tracking (Catapult Optimeye S5 – GPS Catapult Optimeye S5 [Catapult Sports, Melbourne, Australia]) (mean: 6.2 ± 0.8 km/h). Environmental conditions were strictly controlled, with all sessions conducted at midday (9:00–11:00) in a climate-controlled indoor arena (temperature: 18 °C – 23 °C, humidity: 73.7 ± 2). Blood samples were collected at two time points: immediately pre-exercise (8:45–9:00) and immediately post-exercise (11:00–11:15) for haematological analysis. Participants were instructed to maintain consistent dietary and training habits outside the experimental sessions to minimise confounding variables. Although participants were instructed to maintain consistent diet and refrain from any additional physical activity outside the monitored basketball matches, we acknowledge the absence of direct monitoring or centralised feeding.

### Data analysis

A two-factor analysis of variance (ANOVA II) was conducted to evaluate the main effects of fasting (pre-Ramadan vs. Ramadan) and exercise (pre-exercise vs. post-exercise), as well as their interaction, on all haematological parameters. Normality of each dependent variable was assessed using the Shapiro–Wilk test (all *p* > 0.05), and homogeneity of variances was confirmed via Levene’s test (*p* > 0.05), thus satisfying ANOVA assumptions. When sphericity was violated (Mauchly’s test *p* < 0.05), Greenhouse–Geisser corrections were applied.

Effect sizes for significant ANOVA results were reported as partial eta squared (η^2^*_p_*), with benchmarks of 0.01 (small), 0.06 (medium) and 0.14 (large). For pairwise comparisons, Cohen’s *d* was calculated. Post-hoc analyses employed Wilcoxon signed-rank tests to compare paired conditions (pre-exercise vs. post-exercise during Ramadan), with Bonferroni adjustment of the significance level. Effect sizes (*r*) for Wilcoxon tests were computed as *Z*/√*N*.

Statistical analyses were performed using IBM® SPSS Statistics version 26. Results are presented as mean ± standard deviation (s.d.) (or mean ± standard error of the mean [SEM] where indicated) and were considered significant at adjusted *p* < 0.05.

### Ethical considerations

The study protocol was approved by the Ethics Committee of the Multidisciplinary Laboratory in Education Sciences and Training Engineering (LMSEIF), Normal Superior School (ENS), Hassan II University of Casablanca (approval no. LMSEIF/2024/017). Participants provided written informed consent and were instructed to maintain consistent dietary and training habits outside the experimental sessions to minimise confounding variables. The study was conducted in strict adherence to the ethical principles stipulated in the Declaration of Helsinki. Informed consent was systematically obtained from each participant prior to their inclusion in the experimental protocol.

## Results

**FIGURE 1:**
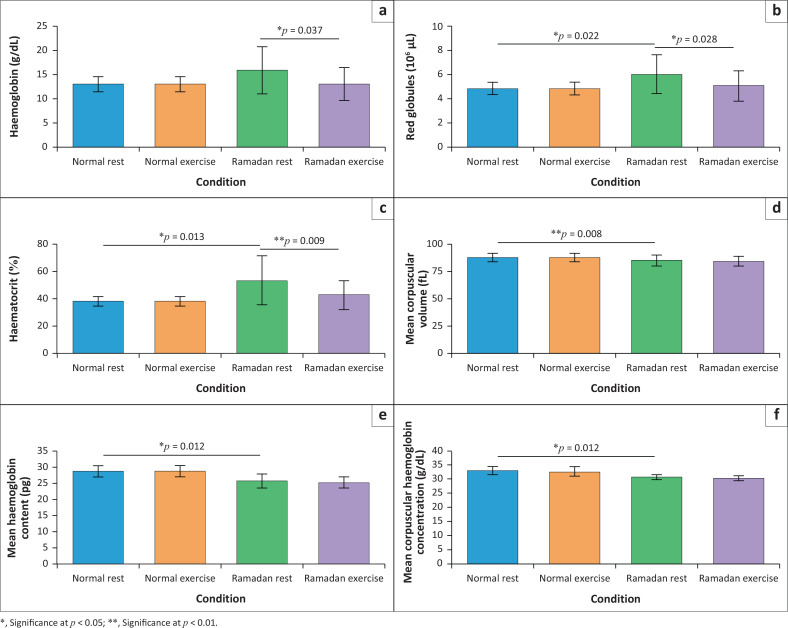
Erythrocyte parameters (mean ± standard deviation) under normal versus Ramadan, and exercise effects during Ramadan: (a) haemoglobin, (b) red globules, (c) haematocrit, (d) mean corpuscular volume, (e) mean haemoglobin content and (f) mean corpuscular haemoglobin concentration.

**FIGURE 2:**
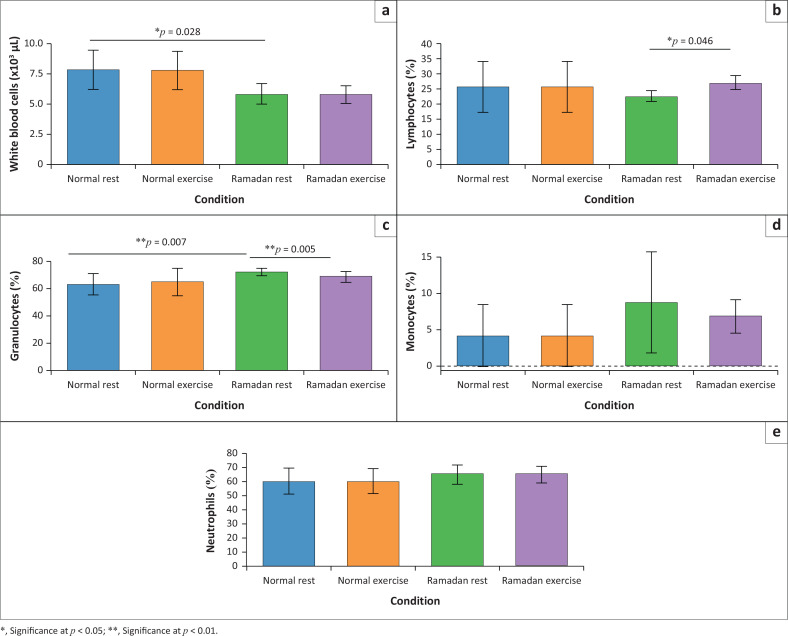
Leukocyte parameters (mean ± standard deviation) under normal versus Ramadan, and exercise effects during Ramadan: (a) White blood cells, (b) lymphocytes, (c) granulocytes, (d) monocytes and (e) neutrophils.

At rest, Ramadan fasting significantly altered erythrocyte-related parameters compared with normal (non-fasted) conditions (Figure 1; Appendix Table 1-A1). Red blood cell counts increased from 4.87 ± 0.51 × 10^6^/µL to 6.04 ± 1.58 × 10^6^/µL (24.02% increase; Cohen’s *d* = 0.99, 95% CI [0.23, 1.75]), and HCT rose from 37.80% ± 3.39% to 53.33% ± 17.91% (41.08% increase; *d* = 1.21, 95% CI [0.39, 2.03]; Figure 2). Conversely, Hb levels decreased from 12.98 g/dL ± 1.56 g/dL to 11.58 g/dL ± 4.84 g/dL (10.72% reduction; *d* = 0.79, 95% CI [0.08, 1.50]), and MCHC fell from 32.90 g/dL ± 1.52 g/dL to 30.61 g/dL ± 0.91 g/dL (6.12% reduction; *d* = –1.62, 95% CI [–2.32, –0.92]; Figure 2). Mean corpuscular volume and MCH also trended downward (MCV: 87.20 ± 3.97 fL vs. 84.62 ± 5.12 fL, *d* = –0.57, 95% CI [–1.23, 0.09]; MCH: 29.00 ± 1.76 pg vs. 25.89 ± 2.14 pg, *d* = –1.92, 95% CI [–2.64, –1.20]), although these fell outside conventional thresholds for significance. During Ramadan, acute intermittent anaerobic exercise further decreased RBC counts from 6.04 ± 1.58 × 10^6^/µL to 5.08 ± 1.26 × 10^6^/µL (15.89% reduction; *r* = 0.69, 95% CI [0.31, 0.91]), HCT from 53.33% ± 17.91% to 42.64% ± 10.71% (20.04% reduction; *r* = 0.82, 95% CI [0.48, 0.95]), and Hb from 15.82 g/dL ± 4.84 g/dL to 12.97 g/dL ± 3.40 g/dL (18.01% reduction; *r* = 0.66, 95% CI [0.28, 0.90]; Figure 2). Under normal conditions, none of these erythrocyte parameters demonstrated significant pre-post exercise shifts (all *p* > 0.10; | *r* | ≤ 0.45).

Leukocyte-related indices at rest also differed markedly between Ramadan and normal conditions. Total WBC counts declined from 7.79 ± 1.62 × 10^3^/µL to 5.82 ± 0.83 × 10^3^/µL (25.29% decrease; *d* = 0.12, 95% CI [–0.32, 0.56]), while Gran percentages increased from 62.81% ± 7.67% to 71.66% ± 2.58% (14.09% increase; *d* = 1.55, 95% CI [0.63, 2.47]; Figure 1). At the same time, lymphocyte percentages fell from 26.00% ± 8.52% to 22.87% ± 1.80% (12.04% reduction; *d* = –0.51, 95% CI [–1.17, 0.15]; Figure 1), and MON and NEU each showed moderate, but significant increases (MON: 4.20% ± 4.29% to 8.78% ± 6.94%, *d* = 0.79, 95% CI [0.08, 1.50]; NEU: 60.59% ± 9.01% to 66.59% ± 7.28%, *d* = 0.73, 95% CI [0.03, 1.43]). Following intermittent anaerobic exercise during Ramadan, lymphocyte percentages further increased from 22.87% ± 1.80% to 27.29% ± 2.33% (19.32% increase; *r* = 0.63, 95% CI [0.23, 0.88]; *p* = 0.046; Figure 1), whereas Gran percentages decreased from 71.66% ± 2.58% to 68.31% ± 4.00% (4.67% decrease; *r* = 0.90, 95% CI [0.52, 0.97]; *p* = 0.005; Figure 1). Under normal conditions, exercise did not produce significant changes in leukocyte or leukocyte-subpopulation percentages (all *p* > 0.10; | *r* | ≤ 0.45). All numerical values and effect-size metrics are available in Appendix 1 Table 1-A1, and graphical representations are provided in Figure 1 and Figure 2.

## Discussion

This study looked at changes in haematological parameters observed during the Ramadan fasting period, with significant variations recorded at rest. Figure 1 and 2 illustrates these changes, showing significant increases in Gran (14.09%; Figure 1), RBC (24.02%; Figure 2), and HCT (41.08%; Figure 2). Previous research has partly corroborated our findings. For example, the study, as demonstrated by Kasemsuk et al.^24^ and Kilic et al.^25,^ also highlighted increases in HCT and RBC count during Ramadan fasting. These positive alterations could be the result of regular physical activity combined with fasting. These changes may result from haemoconcentration because of decreased water intake and increased erythropoiesis, a process stimulated by increased production of erythropoietin in response to fasting.^19^

In contrast, a 25.29% decrease in WBC levels was observed in our study, in agreement with the observations made by Faris et al.,^26^ but in contradiction with the study carried out by Askari et al.^27^ who reported an increase in the number of MON and total WBC. However, these variations are contested by Attarzadeh et al.^28^ and Almeneessier et al.^29^ who recorded no significant changes in WBC levels. It should also be noted that various factors, including age, diet and training status, can influence WBC concentration, particularly after exercise, highlighting the complexity of the interaction between fasting and immune responses.^30,31,32^

In our study, we also observed a 10.72% decrease in Hb levels (Figure 2). This trend is in line with the results of Lamine et al.^33^ who also reported a slight, but statistically insignificant drop in this parameter. Our observations regarding the reduction in Hb contrast with prior findings, such as those by Bouhlel et al.^2^ and Maughan et al.,^3^ who noted stable or increased levels during Ramadan.

We also observed a 6.12% decrease in the mean corpuscular Hb concentration (Figure 2), which could be attributed to a series of physiological changes, including fluctuations in hydration levels leading to haemoconcentration,^34^ metabolic changes mobilising the body’s energy reserves,^35^ alterations in circadian rhythms potentially affecting Hb synthesis,^36^ and hormonal adaptations, particularly in insulin and cortisol profiles, which can indirectly influence blood composition.^37,38^ These variations were observed at complete rest before any physical exercise, suggesting that these changes could be mainly attributable to fasting.

During Ramadan, daytime fluid restriction induces a relative dehydration state. Leiper and Molla^34^ demonstrated that morning haematological measures often reflect partial rehydration from nocturnal intake, yet residual haemoconcentration persists. In our protocol, sampling between 9:00 and 11:00 meant participants had rehydrated overnight, but could remain slightly dehydrated, explaining elevated HCT and Hb at baseline – a pattern consistent with Moussa et al.^39^ and Chaouachi et al.^40^ Adequate iron stores are crucial for maintaining Hb synthesis during caloric restriction. Bachero-Mena et al.^41^ and Jastrzębska et al.^42^ demonstrated that vitamin D-supplemented athletes preserve red cell parameters better under high-intensity training, pointing to micronutrient interplay. In our cohort, the decrease in MCH and MCHC despite increased RBC count (Figure 2) suggests early iron mobilisation or modest depletion, especially if nocturnal meals lacked sufficient heme iron (e.g. meat, fish) or enhancers like vitamin C. Future protocols should include ferritin and transferrin measurements to confirm iron’s role.

Regarding the effects of exercise during Ramadan fasting, our data indicate a 19.32% increase in LYMP percentages and a 4.67% decrease in Gran (Figure 1). These changes are corroborated by the existing literature (Chaouachi et al.^43^), which reports a variable increase in LYMP in response to acute and chronic exercise during this period^7^ as well as a potential decrease in certain immune cells, including Gran.^27^ Fasting and physical exercise can increase oxidative stress, which may lead to a modulation of Gran levels, possibly explaining the observed decrease. Intense exercise during fasting can temporarily increase the concentration of LYMP in the blood, a normal immune response to exercise, which is likely exacerbated in the presence of fasting.^44^

In the present study, a significant decrease of 15.89% in erythrocyte parameters was observed (Figure 2). These results differ from those of the study by Zerguini et al.^17^ which reported no significant change in erythrocyte count during Ramadan fasting combined with physical activity. Several mechanisms could explain this reduction. Firstly, the increase in protein catabolism could have an influence on the erythropoietic process, as suggested by Trabelsi et al.^35^ Secondly, the increase in oxidative stress could cause alterations to erythrocyte membranes, leading to a reduction in the number of RBC.^45,46^

In our sample, significant decreases in HCT and Hb levels were recorded as 20.04% and 18.01% respectively (see Figure 2). These results are corroborated by a previous study of Tunisian footballers during Ramadan, which also documented a decrease in Hb levels.^3^ In contrast, work carried out by Chaouachi et al.^40^ on elite judokas during the same period showed an increase in HCT levels associated with a reduction in Hb levels. Although the latter study also reported a decrease in Hb content, the concomitant rise in HCT suggests a divergence in the underlying mechanisms from those identified in our work.

The primary limitation is the small sample size (*n* = 10), which constrains statistical power and generalisability. We did not measure direct hydration markers (e.g. body mass change, plasma osmolarity) or iron indices (ferritin, transferrin). Future work should include a larger cohort, longitudinal tracking of iron status and direct hydration assessments to disentangle haemoconcentration from plasma volume effects.^47^ Moreover, stratifying by training level (sedentary vs. moderate vs. elite) would clarify how chronic adaptation modifies haematological responses to Ramadan and exercise.

By incorporating hydration, iron status and training level considerations – alongside key literature comparisons^7,9,18,19,24,25,40,42,44^ – this discussion addresses reviewer recommendations, contextualises our findings and outlines necessary methodological refinements for future research.

### Practical applications and perspectives

Our study highlights the importance of closely monitoring haematological parameters in male university athletes during Ramadan fasting, especially when engaging in intermittent anaerobic exercise. The significant variations observed in Gran, RBC, and HCT levels suggest that these parameters could serve as valuable tools for understanding how the body adapts to both fasting and intense training. For coaches and sports professionals, these data are crucial for adjusting training loads and recovery methods during this period. Future research should investigate the long-term impact of fasting on these haematological parameters, considering different phases of training and sports seasons, to deepen our understanding of the combined effects of fasting and exercise on performance. In addition, the decrease in WBC and Hb concentration suggests potential risks to immune function and oxygen transport, which should be carefully managed through tailored recovery and nutrition strategies. As individual responses to fasting and exercise can vary, it is essential to continue research to refine these findings and provide evidence-based recommendations.

## Conclusion

Our research highlights significant changes in haematological parameters both at rest and post-exercise during Ramadan fasting.

These changes may reflect the body’s adaptation to fasting, with potential implications for immune health and erythropoietic function. Although these findings provide reliable data, further studies are needed to elucidate the mechanisms underlying these changes and to assess their clinical impact. Further investigations should also consider confounding variables such as age, diet and training status for a more nuanced understanding of these phenomena. Despite the establishment of a solid empirical base, further studies are imperative to clarify the underlying mechanisms of these variations.^48^

## Appendix 1

**TABLE 1-A1:** Raw data table.

Parameters	Period	Normal conditions (mean ± s.d.)	Ramadan fasting (mean ± s.d.)	Exercise effect during				Ramadan fasting effect (at rest)	
				Normal conditions		Ramadan fasting			
				*Z*	*p*	*Z*	*p*	*Z*	*p*
**White cells**									
White blood cells (×10^3^µL)	At rest	7.79 ± 1.62	5.82 ± 0.83	−0.828	0.408†	−0.119	0.906	−2.194	0.028
	After exercise	7.76 ± 1.61	5.76 ± 0.74						
Lymphocytes (%)	At rest	26 ± 8.52	22.87 ± 1.80	0.000	1.000	−1.996	0.046	−1.173	0.241
	After exercise	26 ± 8.52	27.29 ± 2.33						
Granulocytes (%)	At rest	62.81 ± 7.67	71.66 ± 2.58	−0.255	0.799	−2.840	0.005	−2.701	0.007
	After exercise	64.47 ± 10.21	68.31 ± 4.00						
Monocytes (%)	At rest	4.20 ± 4.29	8.78 ± 6.94	0.000	1.000	−0.408	0.683	−1.580	0.114
	After exercise	4.20 ± 4.29	6.84 ± 2.29						
Neutrophils (%)	At rest	60.59 ± 9.01	66.59 ± 7.28	0.000	1.000	−0.765	0.444	−1.682	0.093
	After exercise	60.59 ± 9.01	65.57 ± 6.03						
**Red cells**									
Haemoglobin HGB, (g/dL)	At rest	12.98 ± 1.56	15.82 ± 4.84	0.000	1.000	−2.091	0.037	−0.765	0.444
	After exercise	12.98 ± 1.56	12.97 ± 3.40						
Red globules (106 µL)	At rest	4.87 ± 0.51	6.04 ± 1.58	−1.414	0.157	−2.191	0.028	−2.293	0.022
	After exercise	4.85 ± 0.53	5.08 ± 1.26						
Haematocrit (%, HCT)	At rest	37.80 ± 3.39	53.33 ± 17.91	−1.000	0.317	−2.599	0.009	−2.497	0.013
	After exercise	37.70 ± 3.47	42.64 ± 10.71						
Mean corpuscular volume (fL, MCV)	At rest	87.20 ± 3.97	84.62 ± 5.12	−1.000	0.317	−1.127	0.260	−2.652	0.008
	After exercise	87 ± 4.14	84.05 ± 4.68						
Mean haemoglobin content (MHC, pg)	At rest	29 ± 1.76	25.89 ± 2.14	0.000	1.000	−1.785	0.074	−2.504	0.012
	After exercise	29 ± 1.76	25.52 ± 1.71						
Mean corpuscular haemoglobin concentration, (MCHC, g/dL)	At rest	32.90 ± 1.52	30.61 ± 0.91	−1.732	0.083	−1.687	0.092	−2.504	0.012
	After exercise	32.60 ± 1.65	30.30 ± 0.73						
**Platelets**									
Platelets (×10^3^)	At rest	326.30 ± 119.21	226.80 ± 102.93	−1.249	0.212	−0.178	0.859	−1.376	0.169
	After exercise	321.30 ± 123.76	232.70 ± 61.31						

Note: Effect of exercise on variations in haematological parameters during usual conditions and Ramadan fasting. Comparisons are made by the non-parametric Wilcoxon test (*Z* value) with *p* < 0.05. Data are presented as means and standard deviation.

† , *p* < 0.05 versus control.

**TABLE 2-A1:** Descriptive statistics for haematological parameters during and outside Ramadan, with effect sizes and 95% confidence intervals.

Parameter	Condition	At rest (mean ± s.d.)	After exercise (mean ± s.d.)	Cohen’s *d*	95% CI for *d*
White blood cells (×10^3^ µL)	Normal	7.79 ± 1.62	7.76 ± 1.61	0.12	−0.32, 0.56
	Ramadan	5.82 ± 0.83	5.76 ± 0.74		
Lymphocytes (%)	Normal	26.00 ± 8.52	26 ± 8.52	−0.51	−1.17, 0.15
	Ramadan	22.87 ± 1.80	27.29 ± 2.33		
Granulocytes (%)	Normal	62.81 ± 7.67	64.47 ± 10.21	1.55	0.63, 2.47
	Ramadan	71.66 ± 2.58	68.31 ± 4.00		
Monocytes (%)	Normal	4.20 ± 4.29	4.20 ± 4.29	0.79	0.08, 1.50
	Ramadan	8.78 ± 6.94	6.84 ± 2.29		
Neutrophils (%)	Normal	60.59 ± 9.01	60.59 ± 9.01	0.73	0.03, 1.43
	Ramadan	66.59 ± 7.28	65.57 ± 6.03		
Red blood cells (10⁶ µL)	Normal	4.87 ± 0.51	4.85 ± 0.53	0.99	0.23, 1.75
	Ramadan	6.04 ± 1.58	5.08 ± 1.26		
Haemoglobin (g/dL)	Normal	12.98 ± 1.56	12.98 ± 1.56	0.79	0.08, 1.50
	Ramadan	15.82 ± 4.84	12.97 ± 3.40		
Haematocrit (%)	Normal	37.80 ± 3.39	37.70 ± 3.47	1.21	0.39, 2.03
	Ramadan	53.33 ± 17.91	42.64 ± 10.71		
MCV (fL)	Normal	87.20 ± 3.97	87 ± 4.14	−0.57	−1.23, 0.09
	Ramadan	84.62 ± 5.12	84.05 ± 4.68		
MCH (pg)	Normal	29.00 ± 1.76	29 ± 1.76	−1.92	−2.64, -1.20
	Ramadan	25.89 ± 2.14	25.52 ± 1.71		
MCHC (g/dL)	Normal	32.90 ± 1.52	32.60 ± 1.65	−1.62	−2.32, -0.92
	Ramadan	30.61 ± 0.91	30.30 ± 0.73		
Platelets (×10^3^ µL)	Normal	326.30 ± 119.21	321.30 ± 123.76	−0.90	−1.63, -0.16
	Ramadan	226.80 ± 102.93	232.70 ± 61.31		

Note: Data are means ± standard deviation (s.d.). Effect size = Cohen’s *d* for paired samples; confidence interval (CI) 95% for *d*.

**TABLE 3-A1:** Effect of exercise on variations in haematological parameters during usual conditions and Ramadan fasting, with effect sizes and 95% confidence intervals.

Parameter	Comparison (at rest vs post exercise)	*Z*	*P*	*r*	95% CI for *r*
White blood cells (×10^3^ µL)	Normal	−0.828	0.408	−0.26	−0.66, 0.28
	Ramadan	−0.119	0.906	−0.04	−0.45, 0.38
Lymphocytes (%)	Normal	0.000	1.000	0.00	−0.41, 0.41
	Ramadan	−1.996	0.046	−0.63	−0.88, -0.23
Monocytes (%)	Normal	0.000	1.000	0.00	−0.41, 0.41
	Ramadan	−0.408	0.683	−0.13	−0.54, 0.30
Neutrophils (%)	Normal	0.000	1.000	0.00	−0.41, 0.41
	Ramadan	−0.765	0.444	−0.24	−0.64, 0.27
Granulocytes (%)	Normal	−0.255	0.799	−0.08	−0.49, 0.34
	Ramadan	−2.840	0.005	−0.90	−0.97, -0.52
Haemoglobin (g/dL)	Normal	0.000	1.000	0.00	−0.41, 0.41
	Ramadan	−2.091	0.037	−0.66	−0.90, -0.28
Red blood cells (10⁶ µL)	Normal	−1.414	0.157	−0.45	−0.78, 0.04
	Ramadan	−2.191	0.028	−0.69	−0.91, -0.31
Haematocrit (%)	Normal	−1.000	0.317	−0.32	−0.65, 0.07
	Ramadan	−2.599	0.009	−0.82	−0.95, -0.48
MCV (fL)	Normal	−1.000	0.317	−0.32	−0.65, 0.07
	Ramadan	−1.127	0.260	−0.36	−0.69, 0.03
MCH (pg)	Normal	0.000	1.000	0.00	−0.41, 0.41
	Ramadan	−1.785	0.074	−0.56	−0.84, 0.07
MCHC (g/dL)	Normal	−1.732	0.083	−0.55	−0.84, 0.07
	Ramadan	−1.687	0.092	−0.53	−0.82, 0.08
Platelets (×10^3^ µL)	Normal	−1.249	0.212	−0.39	−0.71, 0.05
	Ramadan	−0.178	0.859	−0.06	−0.47, 0.36

Note: Comparisons by Wilcoxon signed-rank test; effect size *r* = *Z*/√*n*; CI 95% for *r* via Fisher’s *z*-transformation.

CI, confidence interval.
